# The Unbearable Lightness of Being^1^

**DOI:** 10.3201/eid1606.AC1606

**Published:** 2010-06

**Authors:** Polyxeni Potter

**Affiliations:** Centers for Disease Control and Prevention, Atlanta, Georgia, USA

**Keywords:** Andy Warhol, pop art, Albert Einstein, Ten Portraits of Jews of the 20th Century, emerging viruses, emerging infectious diseases, art science connection, about the cover

**Figure:**
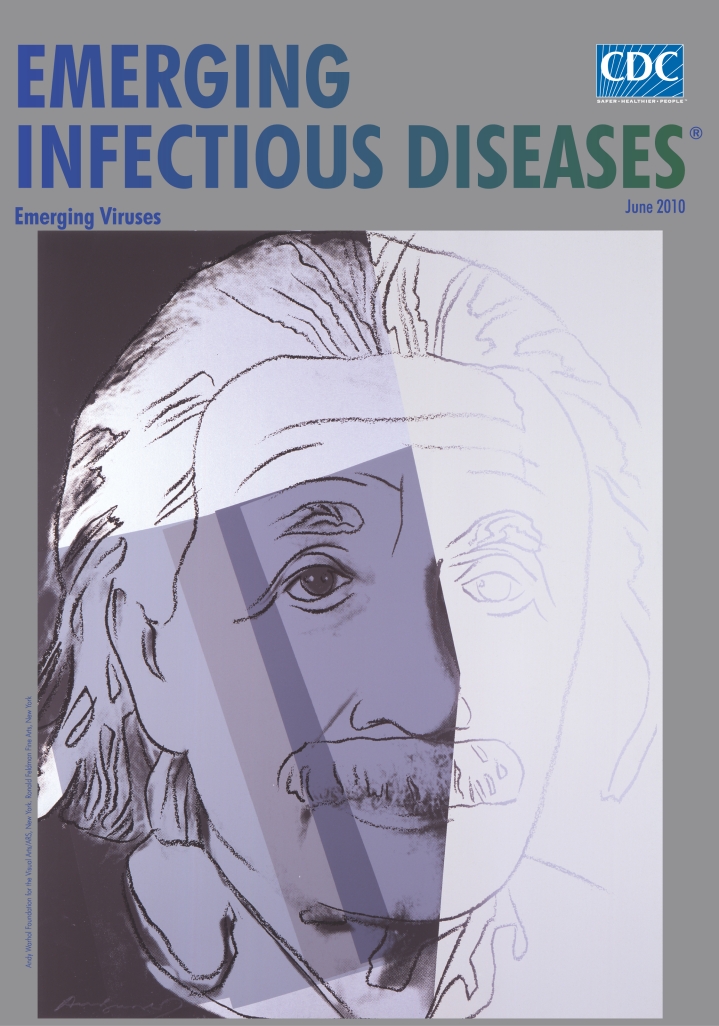
**Andy Warhol (1928–1987) *Albert Einstein* (1980)** Screen Print on Lenox Museum Board (101.6 cm × 81.3 cm) Andy Warhol Foundation for the Visual Arts/ARS, New York. Ronald Feldman Fine Arts, New York, New York, USA

“I’d prefer to remain a mystery. I never like to give my background and, anyway, I make it all up different every time I’m asked,” Andy Warhol said. “It’s not just that it’s part of my image not to tell anything, it’s just that I forget what I said the day before, and I have to make it all up over again.” The man described as “a serious artist whose posture was unseriousness” called himself “a deeply superficial person.” He frustrated those who wanted to know more about him: “Just look at the surface of my paintings and films and me, and there I am. There’s nothing behind it.”

Though he claimed to “come from nowhere,” he was born Andrew Warhola in Pittsburgh, Pennsylvania, to Czech immigrant parents. He lost his father early and grew up in a modest household during the Depression, shy and frail and plagued by childhood diseases, among them Sydenham chorea, a movement disorder that occurs with rheumatic fever, which interfered with his early formal education. As he lay bedridden for months, he listened to the radio; discovered magazines, which he read avidly; and started to draw, entering his artistic career in tune with the popular culture of his generation.

His talent was recognized early. He attended the then Carnegie Institute of Technology in his hometown, to study pictorial design and soon moved to New York to work as commercial illustrator. His first assignment for Glamour magazine was titled “Success Is a Job in New York.” He found such success quickly with other magazines. He designed window displays for major retailers and promotional materials for record labels. Much of this work, which received many accolades, relied on photographs and other existing images, a process he would continue to use throughout his life.

The 1960s saw Warhol’s debut in the fine arts, his Pop paintings, depicting celebrities, dollar bills, and supermarket products. Pop Art, a movement of the 1950s and 1960s, trampled tradition by embracing mass-produced elements of popular culture. Drawn from advertising, comic books, and the mass media and examined in a different light, these mundane elements were transformed into icons of contemplation―product labels and all. The controversial images were created by mechanical techniques of reproduction and established new boundaries for originality and aesthetic legibility. “What about your transformation from being a commercial artist to a real artist?” Warhol was asked in an interview. “I’m still a commercial artist,” he retorted. “I was always a commercial artist.”

Warhol’s *Campbell’s Soup Cans* in 1962, along with his depictions of cartons used for shipping and his sculptures *Brillo Soap Box*, started him on the way to fame. Unlike other Pop artists, who injected their works with irony or parody, he treated them with detachment, emphasizing, even idealizing, mass production. When asked why he painted cans of soup, he said, “I used to drink it. I used to have the same lunch every day for 20 years, I guess, the same thing over and over again.” As for the dollar bills, he explained, “A teacher once told me to paint what I like.”

He opened a studio, dubbed the Factory, and surrounded himself with artists, writers, musicians, and the avant-garde. There he perfected the two-layer screen-printing technique for his portraits. Also called silk-screening, this technique generated a precise image that could mass produce prints—a most appropriate goal considering the duplicative nature of his subjects, who existed not only in life but also on film, in magazines, newspapers, and billboards. Clips from these sources were transferred photographically to the silk or other fine mesh. The canvas was painted, completely or in part, and the screened image, which was slightly larger or smaller than the canvas, was printed on top of the painted designs, allowing some variation in each image.

At the Factory, Warhol created sculptures; published a magazine, The Interview; and managed an electronic rock band called the Velvet Underground. “Your work as an artist has always been so varied, like Leonardo. You’re a painter, a filmmaker, a publisher …. Do you think that’s what an artist is?” He was once asked. “No,” he said, “I think an artist is anybody who does something well, like if you cook well.” At the time of his death he was host of an MTV cable program called “Andy Warhol’s Fifteen Minutes”―a reference to his famous comment that in the future everyone will be world famous for 15 minutes. In the 1960s, 1970s, and 1980s, he produced more than 1,000 portraits, among them, of Marilyn Monroe, Nelson Rockefeller, Elvis Presley, Jackie Kennedy, Brigitte Bardot, and Mao Zedong.

Warhol, always cognizant of the cinematic and telegraphic nature of life, became interested in death and created portraits of people grieving, deceased public figures, the electric chair, car wrecks, race riots. This obsession with death ironically collided with an attempt on his own life inside his studio when a disgruntled disciple shot him and he nearly died. “Before I was shot, I always thought that I was more half-there than all-there. I always suspected that I was watching TV instead of living life. People sometimes say that the way things happen in movies is unreal, but actually it’s the way things happen in life that’s unreal. The movies make emotions look so strong and real, whereas when things really do happen to you, it’s like watching television―you don’t feel anything.”

The philosophical issues surrounding life and death permeated Warhol’s work. “Isn’t life a series of images that change as they repeat themselves?” he said about image repetition in series and within individual works. When he painted soup cans or Coca-Cola bottles, he filled the canvas with them in every direction, suggesting that the line-up did not end at the edge. He insisted that all images are portraits. When he painted a portrait, it was a series.

Repetition, present at many levels in Warhol’s work, is not unique in the history of art. He was aware of its use by Giorgio de Chirico (1888–1978): “He repeated the same images over and over again …. What he repeated regularly, year after year, I repeat the same day in the same painting. All my images are the same, but very different at the same time.” Warhol also cited Edvard Munch: “We see with different eyes at different times. We see things one way in the morning and another in the evening, and the way we view things also depends on the mood we are in. That is why one subject can be seen in so many ways and that is what makes art so interesting.”

In *Ten Portraits of Jews of the 20th Century*, the artist used archival photos over patches of color. He outlined the features in graphite, enlarged the source image on paper, and transferred both onto acetate transparencies. Against convention, the prints preceded the paintings by several months. The same silk-screens were used for multiple prints and paintings, further challenging the divide between painted original and printed reproduction. One of the 10 portraits was *Albert Einstein*, on this month’s cover. Like the others, this is less a portrait and more an icon; the icon of intelligence.

Further repetition and simulacra exist beyond these portraits and other Warhol works. His paintings are a sequence of recurrences already preceded by many and to be followed by many, like the eternal paths encountered by Friedrich Nietzsche’s famed Zarathustra on the mountainside, embodying the philosopher’s discussion of eternal recurrence: the universe and all in it have already occurred and will recur *ad infinitum*, “the most burdensome thought.” Yet each icon reflects the moment of its creation and is original. Thus the artist challenges the nature of seriality as described in antiquity and more recently in the work of Nietzsche and others, who attributed meaning to life by suggesting it was cyclical—the snake bites its tail. The artist was closer to those who disagreed, among them novelist Milan Kundera, who considered the “unbearable” possibility of no recurrence at all. At death, each person, each copy is gone.

Warhol understood how the media shape our view of the world by appropriating and projecting icons, a process he adopted and used to great advantage. Along with another media favorite, repetition, this “deeply superficial” artist captured and held up for us to see, as in a mirror, the media’s fragmented version of reality. His technique fit perfectly the portrait of Einstein, whose expansive vision also allowed more than one version of reality.

With characteristic unseriousness, Warhol proposed a more contemporary view of eternal recurrence: originality in duplication. This approach has a parallel in nature, which in this issue of Emerging Infectious Diseases relates to virus replication. A virus inserts itself into the cellular machinery, which produces and reassembles new viral particles—an age-old serial activity that will continue to recur. Like Warhol’s portraits, the replication is not always completely true, so some copies are not identical. The next virus, even though a replica, is not the same. The defining differences can be beneficial for virus replication and survival and may favor the emergence of infectious disease.
